# Polarizable *ab initio* QM/MM Study of the Reaction Mechanism of *N*-*tert*-Butyloxycarbonylation of Aniline in [EMIm][BF_4_]

**DOI:** 10.3390/molecules23112830

**Published:** 2018-10-31

**Authors:** Erik Antonio Vázquez-Montelongo, José Enrique Vázquez-Cervantes, G. Andrés Cisneros

**Affiliations:** 1 Department of Chemistry, University of North Texas, Denton, TX 76201, USA; erikvazquezmontelongo@my.unt.edu (E.A.V.-M.); enriquevazquezcervantes@my.unt.edu (J.E.V.-C.); 2 The Center for Advanced Scientific Computing and Modeling (CASCaM), University of North Texas, Denton, TX 76201, USA

**Keywords:** QM/MM, minimum energy path, topological analysis, non–covalent interactions

## Abstract

*N*-tert-butoxycarbonylation of amines in solution (water, organic solvents, or ionic liquids) is a common reaction for the preparation of drug molecules. To understand the reaction mechanism and the role of the solvent, quantum mechanical/molecular mechanical simulations using a polarizable multipolar force field with long–range electrostatic corrections were used to optimize the minimum energy paths (MEPs) associated with various possible reaction mechanisms employing the nudged elastic band (NEB) and the quadratic string method (QSM). The calculated reaction energies and energy barriers were compared with the corresponding gas-phase and dichloromethane results. Complementary Electron Localization Function (ELF)/NCI analyses provide insights on the critical structures along the MEP. The calculated results suggest the most likely path involves a sequential mechanism with the rate–limiting step corresponding to the nucleophilic attack of the aniline, followed by proton transfer and the release of CO${}_{2}$ without the direct involvement of imidazolium cations as catalysts.

## 1. Introduction

Ionic liquids (ILs) are molten salts composed of (usually) organic cations and inorganic or organic anions. An interesting feature of many of these cation/anion mixtures is that they are liquid at room temperature (RTIL) [1,2]. The very large number of available combinations of anions and cations (and their structural features) results in a myriad variations of physicochemical properties, such as relatively low viscosity [3], virtually nonexistent vapor pressure, high thermal conductivity, to name a few [1,2,3,4]. ILs have been use in a wide range of applications, such as electrochemistry [5] (nonaqueous electrolyte) and for applications in synthetic chemistry. Recently, these liquids have been used as an alternative for organic solvents in enzymatic catalysis [6,7,8].

ILs provide an attractive alternative to organic solvents for several applications due to their immiscibility with many solvents (even with water), and because they are nonvolatile, nonflammable, and thermally stable liquids [3,9,10,11]. Additionally, their exceptional solvation and dissolution properties make them useful solvents for homogeneous catalysis [3,12,13]. One particular example involves the protection reaction of primary amines. Sarkar et al. have proposed that ILs based on 1-alkyl-3-methylimidazolium may act as catalysts for the efficient *N*-*tert*-butoxycarbonylation of amines with good chemoselectivity [14]. However, other studies have proposed that this reaction proceeds via a catalyst-free mechanism [15,16].

Computational simulations have become a useful tool to investigate IL properties given the very large number of possible combinations. Simulations based on classical molecular dynamics using nonpolarizable point charges have shown to provide insights into these systems [17,18,19,20,21,22,23,24]. It has been shown that the inclusion of polarization can improve the description of IL-based systems [25,26,27,28,29,30]. Some of us have extended AMOEBA to investigate a variety of IL systems [31,32,33,34,35,36,37]. AMOEBA is a multipolar/polarizable potential that relies on distributed atomic multipoles up to quadrupoles and inducible point dipoles [38,39,40]. We have shown that distributed multipoles obtained fitting electronic densities via the Gaussian electrostatic model (GEM) can provide accurate multipoles for several systems, including ILs [31,41,42,43].

Computational simulations have also been employed to investigate reaction mechanisms of chemical reactions in ILs using quantum-mechanics/molecular-mechanics (QM/MM) simulations [44,45,46,47,48,49,50]. Nonpolarizable force fields (NPFF) such as OPLS [51], CHARMM [52,53], AMBER [54,55], or GROMOS [56], have been typically used to model the classical environment in the QM/MM system, and to study solute–solvent interactions, conformational changes, and reaction mechanisms in ILs and other types of systems, such as biological and condensed-phase systems [57,58,59,60,61,62,63,64,65,66,67,68,69,70,71,72,73,74].

Polarization has also been shown to be important in QM/MM simulations [29,75,76,77,78,79,80,81]. However, to our knowledge, there have been no QM/MM simulations of reactions in IL systems that include an explicitly polarizable MM environment. In this contribution, we present the use of AMOEBA-based polarizable ab initio QM/MM simulations to investigate the reaction mechanism of the *N*-*tert*-butoxycarbonylation of aniline (base reaction for the preparation of drug molecules [82]) in a water/1-ethyl, 3-methyl imidazolium/tetrafluoroborate ([EMIm][BF${}_{4}$]) mixture. Complementary combined electron localization function (ELF)/noncovalent interaction (NCI) [83,84,85,86,87,88,89,90] analyses were used to gain insights on the solvent–solvent, solvent–solute, and solute–solute intra- and intermolecular interactions.

The remainder of the paper is organized as follows: in the next section, we present the computational methodology for the calculation of the required AMOEBA parameters, details of the QM/MM and QM simulations for the reference gas-phase and implicit-solvent reaction, as well as the corresponding cross ELF/NCI analyses. Subsequently, we present the results for the considered reaction mechanisms, followed by concluding remarks.

## 2. Methodology

This section presents the details of the computational methods and procedures employed for the parametrization of aniline and di-*tert*-butyl dicarbonate [(Boc)${}_{2}$O] for AMOEBA, followed by a description of the QM methods and the procedure for the QM/MM simulations using LICHEM for the determination of the reactants and products, as well as the minimum energy paths (MEPs).

### 2.1. Parametrization

AMOEBA parameters for [EMIm][BF${}_{4}$] have been previously reported [34,35]. The parameters for aniline and di-*tert*-butyl dicarbonate molecules for AMOEBA [38,39,40] were obtained following the methodology previously described [32,33,34,40]. The bonded parameters were taken from available parameters for similar molecules without any adjustment [40]. The nonbonded (electrostatic, polarization, and van der Waals) parameters were fitted based on QM energy-decomposition analysis (EDA) [32,33,34]. Briefly, the EDA-based nonbonded parametrization procedure was performed as follows: (1) calculation of basis set superposition error (BSSE)-corrected total intermolecular energies between aniline/[(Boc)${}_{2}$O] and water dimers oriented randomly at the ωB97XD/aug-cc-pVTZ level of theory using the Gaussian09 software package [91]; (2) computation of the electrostatic and polarization contributions for the same dimers using Symmetry-Adapted Perturbation Theory (SAPT) with Psi4 [92], at the SAPT0/6-311+G(d,p) level of theory; (3) calculation and optimization of GEM distributed multipoles (GEM-DM) using GEM-fit [31] by minimization of the Coulomb intermolecular interaction error with respect to the SAPT Coulomb reference.

### 2.2. Molecular-Dynamics Simulations

Molecular-dynamics (MD) simulations of a mixture of 7410 water molecules and 189 [EMIM][BF4] ion pairs, corresponding to a 2.5% mol solution of IL, were performed using AMOEBA [38,39,40], employing the TINKER–OpenMM software package [93]. The system was equilibrated for 1 ns using the RESPA integrator, 2 fs time step, and the Bussi thermostat. Subsequently, one molecule of aniline and one molecule of [(Boc)${}_{2}$O] were placed in the center of the equilibrated water/IL mixture. The closest cation and anion pair to the solute molecules was manually repositioned to within 3 Å of the solvent molecules. The resulting solute/solvent system was subjected to a 1 ns MD with a distance constraint of 15 (kcal/mol)/Å${}^{2}$ between the solute and nearest ion pair molecules. Finally, the distance constraint between the aniline/[(Boc)${}_{2}$O] and the ionic pair was removed and the system was subjected to unrestrained MD for 1 ns.

### 2.3. Gas-Phase and Implicit Solvent Calculations

The critical structures (reactants, products, intermediates, and transition states) for each of the tested schemes were optimized in the gas phase and in implicit solvent (CH${}_{2}$Cl${}_{2}$) at the ωB97XD [94] level of theory with the 6-31G(d) [95] basis set using Gaussian09 [91]. For transition state optimization, the Synchronous Transition Quasi-Newton (STQN) method [96] was used (implemented as QST3 in Gaussian09). Frequency calculations for reactant, intermediate, product, and transition state were performed. All thermochemistry calculations were carried out at STP conditions.

### 2.4. QM/MM Geometry and Reaction-Path Optimizations

All QM/MM calculations were performed using the LICHEM software package [81]. The QM region (aniline + di-*tert*-butyl dicarbonate + one IL pair + water) was calculated at the ωB97XD level of theory using the 6-31G(d) basis set. Two QM/MM simulation systems were created based on the above MD simulations, one with the ion pair close to the solute (C1), and one after the constraints had been released (C2). The QM regions for both systems were selected based on the number of molecules around an 8 Å sphere with respect to a central C on the (Boc)${}_{2}$O molecule (see below). In all cases, the QM/MM systems did not involve covalent bonds across the QM/MM regions. The MM region (26688 atoms) was modeled using the AMOEBA force field. Long-range electrostatic (LRE) effects were taken into account using LRE correction (LREC) approximation [97,98], with an LREC exponent of 2 and a cutoff radius of 20 Å.

Path optimizations for the QM/MM systems were performed by means of two chain-of-replica methods, the nudged elastic band (NEB) or quadratic string method (QSM) [99,100]. A linear interpolation between the reactant and product structures was performed using 17–19 beads to generate the initial guess for the replicas along the chain using the path utility in LICHEM. For the NEB optimizations, 20 path optimization steps and 20 QM optimization steps were used for the geometry and reaction-path calculations using a spring constant of 1 eV/Å${}^{2}$. The QM tolerance was set to a RMS deviation of 0.001 Å, RMS force of 0.005 Hartree/Bohr, and maximum RMS force to 0.02 Hartree/Bohr. MM tolerance was set to a RMS deviation of 0.2 Å and a RMS force of 0.2 kcal/Å.

In some cases, it has been observed that the use of a second-order path optimizer such as QSM can provide a way to refine or overcome convergence issues for first-order path optimization methods [72,101]. We have recently implemented QSM in LICHEM [100,102]. In QSM, each replica on the MEP is perpendicularly minimized to the reaction coordinate direction through a quadratic surface approximated by a damped Broyden–Fletcher–Goldfarb–Shanno (BFGS)-updated Hessian, followed by an N-order Runge–Kutta adaptive step-size integration method.The same settings/tolerances as above, for the NEB paths, were employed for the QSM-optimized paths. Both NEB and QSM lack explicit TS optimizers, and thus the highest energy points obtained from the optimized MEPs are only approximations of the corresponding TS [99,100].

### 2.5. Combined ELF/NCI Analyses

All wave functions for the ELF and NCI calculations were obtained using the LICHEM software package [81], with the same level of theory used for the QM/MM calculations. ELF calculations were computed with the ToPMoD software package [103,104], using a cubic grid of 200 a.u. with a step size of 0.1 a.u., with an isovalue of 0.83. For the NCI calculations, the NCIPLOT software package [87] was used, with an isovalue of 0.5 au with the color scale with a range of −0.05 au < sign(λ2)ρ < 0.05 au.

## 3. Results and Discussion

### 3.1. Parametrization Results

The GEM-distributed multipoles (GEM-DM) for the solute molecules were obtained by fitting electronic densities at the DFT level for each molecule as described above. Molecular densities were optimized by minimizing the error of the intermolecular Coulomb interaction between the fitted density of the target molecule and a water molecule with respect to the Coulomb component from SAPT0 for a set of solvent/water dimers in random orientations to ensure transferabitliy [34,35,58].

**Figure 1:**
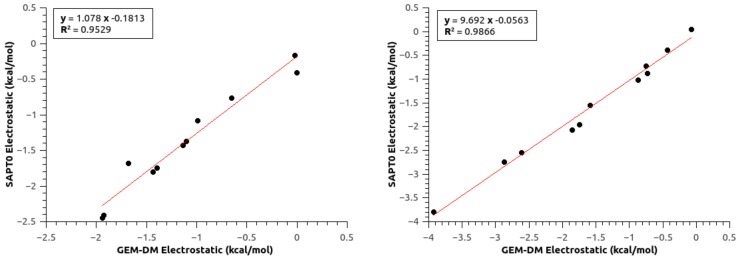
Intermolecular Coulomb interaction for the set of randomly oriented aniline/water (**left**) and [(Boc)${}_{2}$O]/water (**right**) dimers.

The comparison between the electrostatic contribution from SAPT0 calculations and the GEM-DM is shown in Figure 1. Previous studies have shown that intermolecular electrostatic interactions calculated with GEM-DM are well described at medium and long range [32,33,34], and deviations arise at short intermolecular distances due to penetration errors [105].

The Van der Waals parameters were obtained by fitting the corresponding contributions by a subtractive approach using the BSSE-corrected total intermolecular interaction energies and SAPT0-calculated components, and comparing with the respective AMOEBA/GEM-DM counterparts [34,35,58]. The total intermolecular interactions (BSSE-corrected and SAPT0) show good agreement with the final set of parameters (see Figures S1 and S2).

### 3.2. Optimizations of Critical Points

**Scheme 1:**
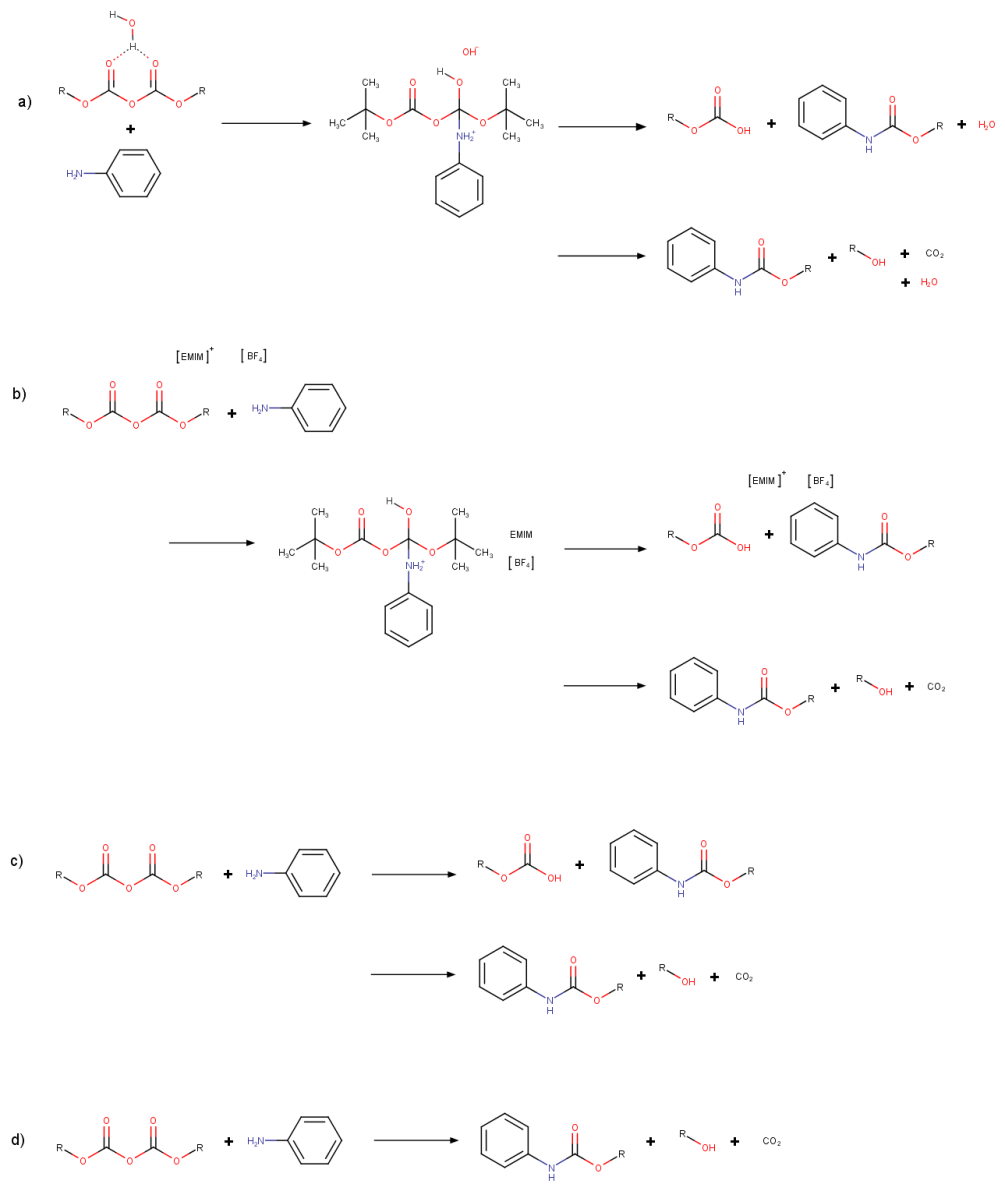
Reaction scheme for the *N*-tert-Butyloxycarbonylation of aniline. Panels (**a**,**b**) show the activation of (Boc)${}_{2}$O through bifurcated hydrogen bond formation between the water molecule or the IL cation ([EMIM]${}^{+}$). Panel (**c**) corresponds to a sequential reaction mechanism without (Boc)${}_{2}$O activation. Panel (**d**) also corresponds to a concerted mechanism.

Four different reaction path possibilities were tested for the *N*-tert-butyloxycarbonylation of aniline based on previously proposed mechanisms (Scheme 1) [14,15]. The first two possibilities (Scheme 1a,b) involve the activation of (Boc)${}_{2}$O through the formation of a bifurcated hydrogen bond between a water or cation ([EMIM]${}^{+}$) molecule, respectively. This step is followed by a nucleophilic attack by the aniline N, formation tert-butyl carbamate and tert-butyl carboxylic acid, and, finally, the release of CO${}_{2}$ from this acid and formation of tert-butanol.

The remaining two proposed mechanisms, shown in Scheme 1c,d, involve a possible sequential path without the involvement of an explicit activating molecule for (Boc)${}_{2}$O. Depending on which O atom from (Boc)${}_{2}$O accepts the leaving proton from the aniline, the formation of CO${}_{2}$ can proceed with or without the formation of tert-butyl carboxylic acid (Scheme 1c,d, respectively).

**Figure 2:**
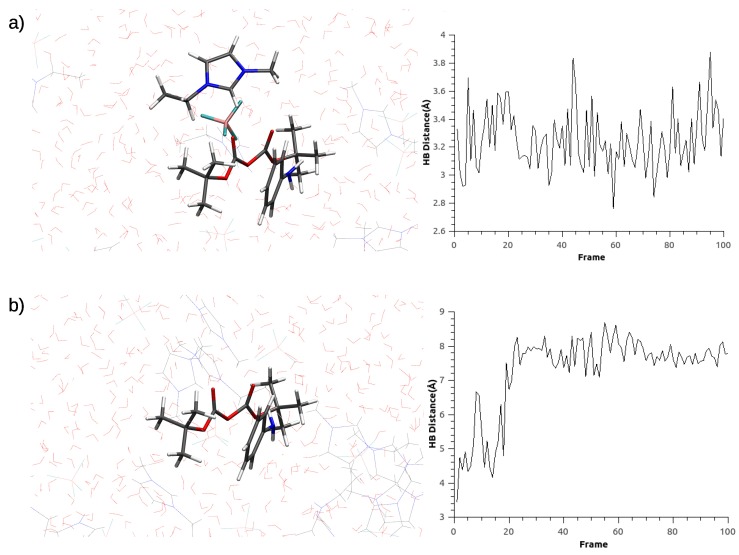
Comparison between the relative distance the reactants and [EMIM][BF${}_{4}$]. (**a**) Molecular dynamics (MD) with restrain distance between reactants and [EMIM][BF${}_{4}$]. (**b**) MD without restrain distance between reactants and [EMIM][BF${}_{4}$]. Where colors correspond to: C–gray, O–red, N–blue, H–white, B–pink, F–cyan.

Based on the mechanistic possibilities described above, two different QM/MM systems were studied based on two configurations of an IL ion pair. The first configuration (C1) was obtained by optimizing a series of snapshots from the MD simulation where the ion pair distance was restrained (Figure 2a) by selecting 149 atoms to be located in the QM region (see Section 2.4). The second configuration, termed C2, was obtained based on the QM/MM optimization of a series of snapshots from the unrestrained MD simulation (Figure 2b) by selecting 155 atoms to be located in the QM region (see Section 2.4). In particular, in C1, the IL cation forms an H-bonding interaction in between both oxygens from the [(Boc)${}_{2}$O] carbonyl groups, whereas in C2, there are no specific interactions with [(Boc)${}_{2}$O]. These two configurations were subsequently employed to test all four mechanistic possibilities described above by optimizing the reactant and product structures at the QM/MM level for all four possible mechanisms in Scheme 1.

The calculated reaction energies for the reactions in Scheme 1a,b are highly endothermic (Table 1). Therefore, these two mechanistic possibilities were not further considered. Conversely, the reaction energies for Scheme 1c,d in the water/IL solution were found to be exothermic.

Geometry optimizations in gas phase (GP) and in dichloromethane (DCM) using the SMD implicit solvent model were performed for structures in Scheme 1c,d for comparison purposes. In all cases, the calculated reaction energies were found to be exothermic; for Scheme 1c, the DCM(GP) reaction energies were −16.11(−17.26), compared with −9.53(−0.19) for Scheme 1d. These results suggest that the water/IL mixture provides significant stabilization of the product structures compared to GP or pure organic solvent (as represented with an implicit solvent model).

### 3.3. Minimum Energy Path Optimizations

The two possible mechanisms (Scheme 1c,d) were further investigated in the water/IL QM/MM systems by determining the minimum energy paths connecting the reactant and product structures. Four MEPs were tested to investigate the reaction path for each possible scheme with either configuration 1 (C1, nearby IL ion pair) or configuration 2 (C2). As described above, Scheme 1c corresponds to the nucleophilic attack without the simultaneous formation of CO${}_{2}$, while Scheme 1d includes the concomittant formation of CO${}_{2}$ after the formation of the carbamate.

**Figure 3:**
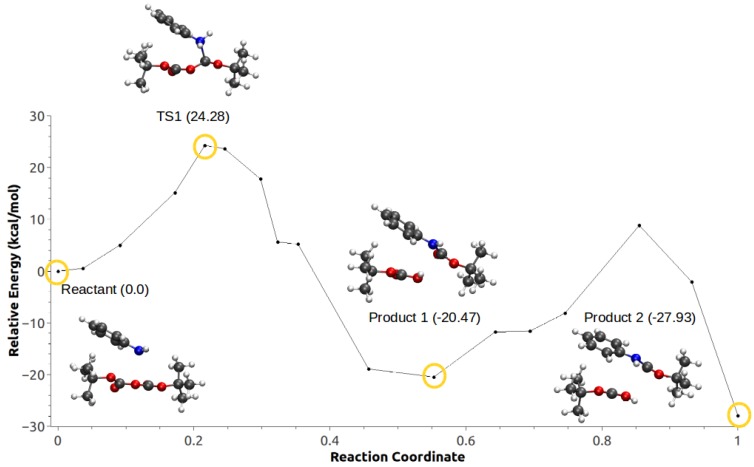
Minimum energy path for Configuration C1, Scheme 1c.

The calculated MEP for Scheme 1c using Configuration C1 (Figure 3 and Video S1) suggest that this path proceeds through an SN${}_{2}$-like mechanism, starting with the nucleophilic attack by the aniline to the carbonyl of one of the Boc groups and the concommitant bond breaking of an O–C bond in Boc–O–Boc (transition state). This is followed by a proton transfer from the amine to the Boc group, and the corresponding formation of the tert-butyl carbamate and tert-butyl carboxylic acid. The highest point on the path has an energy barrier of 24.28 kcal/mol with respect to the reactant structure. Two products were observed along with this MEP, which correspond to two different orientations of the hydroxyl H formed after the proton transfer from the aniline. The final product (Product **2** in Figure 3) was around 8 kcal/mol lower in energy than the initially optimized product.

**Figure 4:**
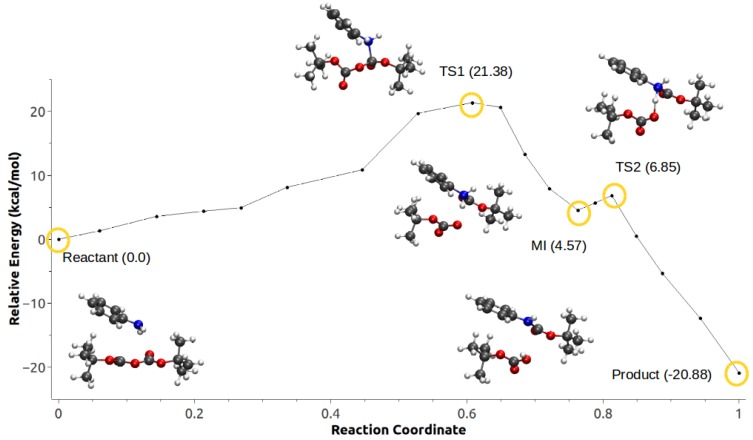
Minimum energy path for Configuration C2, Scheme 1c.

A similar reaction path to the one calculated for Configuration C1 was found for Scheme 1c using Configuration C2 (Figure 4 and Video S2). The major differences between the two configurations involve a reduction of around 3 kcal/mol in barrier height for C2 compared with C1, and no observed rotation of the H bond to form a second product. Interestingly, the change in the anisotropic field by having the IL ion pairs further away resulted in the stabilization of a metastable intermediate (MI) corresponding to the product of the nucleophylic attack, but prior to the proton transfer, coupled with a second low-barrier TS associated with the proton transfer, and leading to the formation of the product. The resulting product structures for both configurations (product **1** for Figure 3) showed very similar geometries and relative energies (C1: ΔE = −20.46 kcal/mol, C2: ΔE = −20.88 kcal/mol).

**Figure 5:**
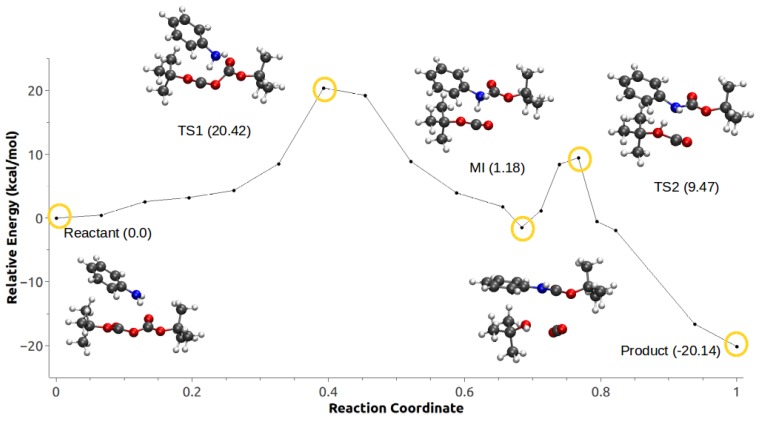
Minimum energy path for Configuration C2, Scheme 1d.

In the case of Scheme 1d, path optimization for the system corresponding to Configuration C1 failed to converge with both NEB and QSM. In both cases, the path exhibited several discontinuities, which could be due to the close proximity of the highly charged ion pair. The QSM-optimized MEP for Scheme 1d with Configuration C2 is presented in Figure 5 (and Video S3). The rate-limiting step had a barrier of 20.42 kcal/mol, which corresponds to the nucleophilic attack of the aniline to the carbonyl of one of the Boc groups, followed by a metastable intermediate and a second energy barrier (1.18 kcal/mol and 9.47 kcal/mol, respectively) corresponding to the proton transfer from the amine to the Boc group, and the subsequent formation of the tert-butanol, CO${}_{2}$, and tert-butyl carbamate.

**Figure 6:**
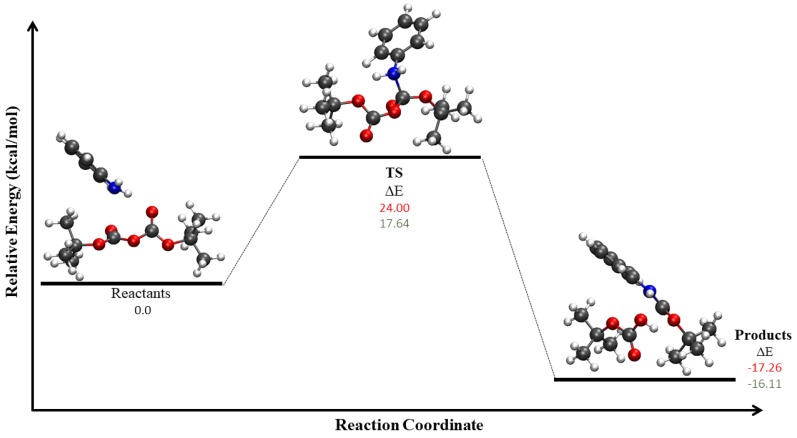
Energy profile for Scheme 1c for gas phase (GP) and dichloromethane (DCM). The figure shows the reaction energy and reaction barrier in the gas phase (red), and with DCM using the SMD implicit solvent model (green).

For the GP and DCM calculations, TS optimizations were performed using the QST3 method [96]. The calculated energy barriers for Scheme 1c corresponded to 24 kcal/mol and 17.64 kcal/mol, respectively (Figure 6). For Scheme 1d, optimization failed to yield TS structures that connect the reactant and product valleys for both the GP and DCM systems.

Previous experimental reports have suggested that this reaction shows very low yields in DCM, and high yields with good stereoselectivity in ILs [14]. The present DCM results suggest the availability of a path to form the protected aniline, but we were unable to find a viable route to the final products, including formation of CO${}_{2}$, via either Scheme 1c or d. Conversely, for the water/IL mixture, the path optimization results suggested Scheme 1d was the most likely mechanism.

### 3.4. Population and Topological Analyses for Critical Structures

**Figure 7:**
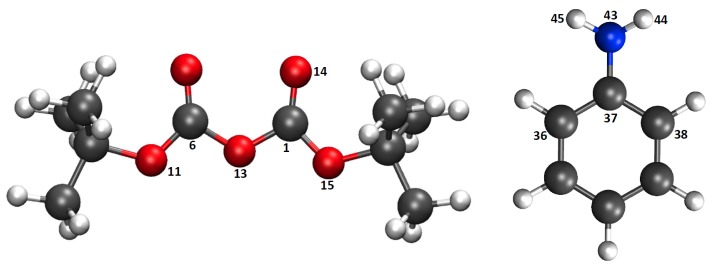
Atom labeling for Merz–Singh–Kollman (ESP) and electron localization function (ELF)/noncovalent interaction (NCI) analysis. Atom coloring is same as in Figure 2.

**Figure 8:**
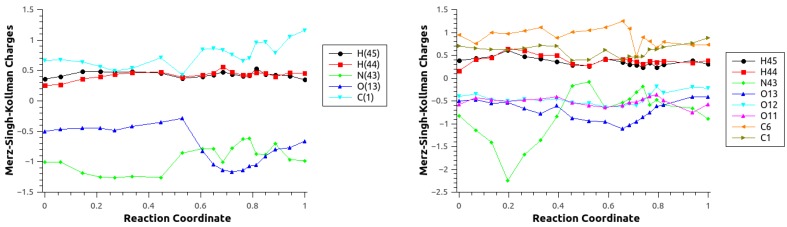
ESP charges for the atoms involved in the MEP for Scheme 1c (**right**) and d **left**) for Configuration C2.

Complementary topological and population analysis has been carried out to gain further insights on the electronic structure and its evolution for critical points along the path. The ESP charge population analysis for the atoms involved in the reaction (Figure 7) for the reaction mechanisms using configuration C2 are presented in Figure 8 for Scheme 1c,d (see Figure S4 for configuration C1).

In the case of Scheme 1c, an increase in charge around the TS was observed for the four atoms involved in the bond-formation/-breaking processes. In particular, atoms N(43) and C(1) show an increase in charge, which is anticorrelated with a decrease in charge for O(13). These results are consistent with the changes in charge observed for the GP and DCM critical points (see Table 2). For Scheme 1d, a significant change in the nitrogen charge was also observed, although the charge appeared to be distributed among the remaining atoms in the system, and not only the atoms directly involved in the bond-breaking/-forming processes.

For the reaction in GP and DCM for Scheme 1c (Table 2), the results were consistent with the ones in the water/IL mixture. Similarly, there was a decrease of the charge for O(13) and an increase for N(43). This behavior could be due to the presence of two ionic species in the transition state (Boc-O${}^{-}$ and Boc-Ph-NH${}_{2}$${}^{+}$), a result of the aniline nucleophilic attack.

**Figure 9:**
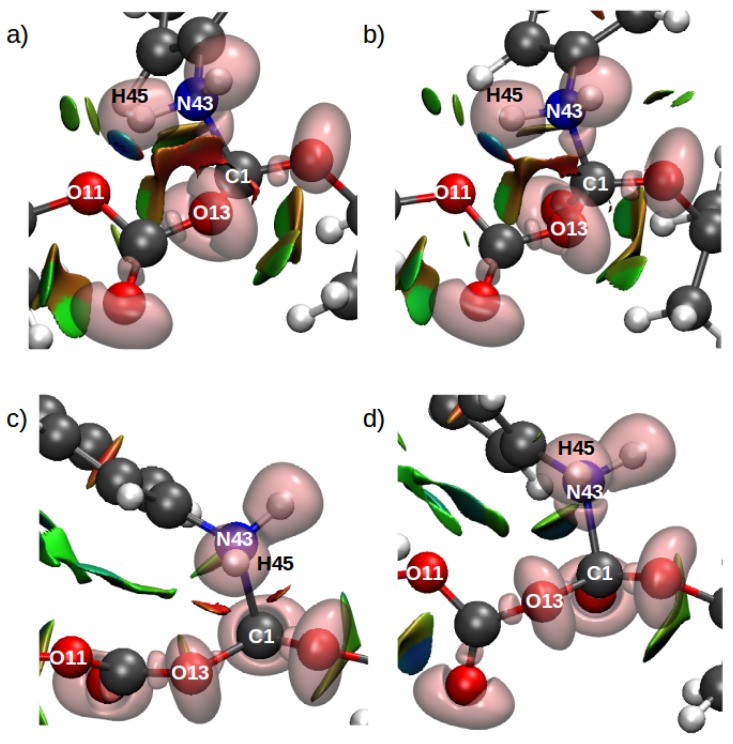
Combined ELF/NCI surfaces for the TS structures for the rate-limiting step in Scheme 1c. (**a**) GP, (**b**) DCM, (**c**) Configuration C1, (**d**) Configuration C2. The isovalue for ELF was 0.83, and for NCI it was 0.5, with a color scale of −0.05 au < sign(λ2)ρ < 0.05 au.

Combined ELF/NCI analyses have been used to provide better insights into chemical bonding and weak noncovalent interactions, as well to understand reaction mechanisms in organic and biological systems [58,64,88,89,90]. The combined ELF/NCI analyses for the TS structures for the rate-limiting step in Scheme 1c for all tested systems (GP, DCM, and Configurations C1 and C2) are shown in Figure 9 (see Figures S5–S8 for all ELF/NCI analyses for all critical structures).

The TS structures for Scheme 1c in all four systems show a disynaptic basin between C1 and N43 in the ELF region between these atoms (pink transparent surface in Figure 9 and Table 3), which corresponds to the formation of the covalent bond resulting from the nucleophilic attack of the aniline on the carbonyl C of Boc. That is, in all cases, the TS structure corresponding to this nucleophilic attack already involved a bond between the N and C atoms, which would suggest that these structures corresponded to a late TS.

In addition, a strong hydrogen bond (H–bond) interaction was observed between H(45) and O(11) for the TSs in the gas-phase and dichloromethane systems (depicted as a large dark-blue surface in Figure 9a,b). Conversely, the TS structures corresponding to the water/IL solution did not exhibit this strong H–bond, which was due to the change in the nucleophilic attack orientation of the aniline due to the stabilization of the surrounding solvent.

The results of ELF population and distributed multipole analyses for the selected basins are presented in Table 3 (see Tables S1–S5 for full multipolar analysis). Overall, the results were in agreement with the corresponding ESP charge population analysis. In particular, a disynaptic basin was observed, V(C1,N43), corresponding to the nucleophilic attack surface discussed above. Interestingly, the population and first polar moment for this basin in TS1 for the reaction in the water/IL mixture was considerably larger than in GP and DCM. In addition, for the water/IL mixture, a monosynaptic basin for the transferring proton was observed, V(H45). This basin was indicative of a strong H–bond interaction. These basins were previously shown to indicate strong interactions, such as low-barrier hydrogen bonds.

**Figure 10:**
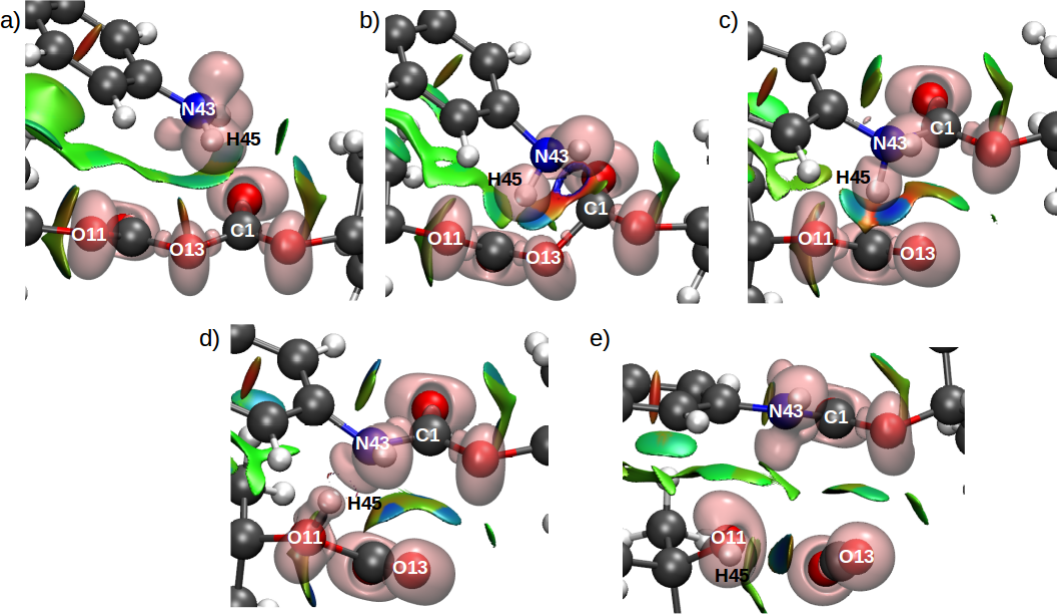
Combined ELF/NCI surfaces of the critical structures for Configuration C2 for Scheme 1d. Panels (**a**–**e**) show the reactants, TS1, MI, TS2, and products structures, respectively. The isovalue for ELF was 0.83 and for NCI was 0.5, with a color scale of −0.05 au < sign(λ2)ρ < 0.05 au.

By contrast, the TS structure for Scheme 1d did not exhibit a disynaptic basin between the attacking N and C atoms. Instead, a ring was observed on the NCI surface in Figure 10b, indicating a strong attractive interaction, although no basin was observed in the ELF analysis. These results suggest that the solvent environment for Scheme 1d stabilized an early TS. Additionally, Figure 10c shows a bifurcated H–bond interaction between H45, O11, and O13, which was resolved in TS2 with the transfer of H49 to O11 (Table 4). Interestingly, no NCI surfaces were observed between the H(45) and N(43), in the TS2, or the product structure, indicating an increase in distance between the resulting product molecules.

### 3.5. Effect of Polarization on the Reaction Path

**Figure 11:**
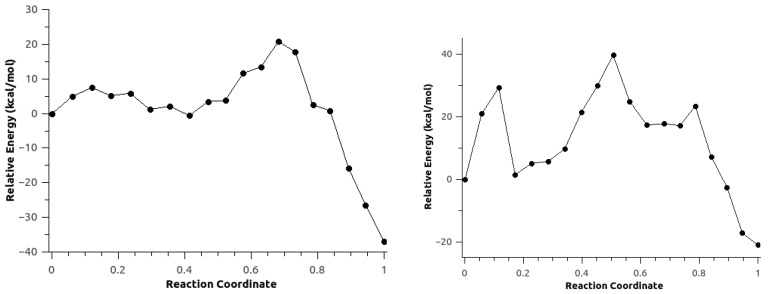
Minimum energy path for Scheme 1c (**right**) and d (**left**) for Configuration C2 without the AMOEBA polarization term.

The MEPs for Scheme 1c,d for Configuration C2 were reoptimized without explicit polarization to investigate the role of polarization of the environment on the reaction path (Figure 11). For Scheme 1c, the resulting energy profile was similar to the one with the use of explicit polarization (Figure 4), with an energy barrier of 21.43 kcal/mol and a reaction energy of −36.87 kcal/mol. However, this MEP lacked the second TS that corresponds to the proton transfer. For Scheme 1d, the energy profile showed some irregularities due to the rearrangement of environmental solvent molecules (see Video S4 for Scheme 1c, Configuration C2; and Video S5 for Scheme 1d, Configuration C2 for supporting information), an energy barrier of 39.67 kcal/mol (almost twice the value for the MEP shown in Figure 5), and a reaction energy of −21.01 kcal/mol. The similarities observed between the energy profiles with and without polarization may be due to the fact that path optimization was performed using the optimized path with polarization as an initial guess for the unpolarized MEP optimization, and the fact that the optimized path corresponded to the potential energy surface, and not the free energy surface. Previously, we showed the importance of the inclusion of explicit polarization of the MM environment due to the QM region, and the polarization of the QM region due to the static field [81], and the importance of polarization in classical simulations on a neutral but highly polarizable ligand [106].

The ESP charges for selected atoms in the TS1 for both Scheme 1c,d (see Figures S9 and S10) indicate a charge redistribution in the neighboring atoms of C1 and N43, and there was a large difference in the charge of the aniline nitrogen (N43) when polarization from the MM environment is considered or not. Inclusion of thermal and entropic effects (e.g., by FEP) would be useful to provide further insights to understand these effects.

## 4. Conclusions

The reaction mechanism for the *N*-tert-butyloxycarbonylation of aniline in a water/IL mixture was investigated by means of polarizable QM/MM calculations. Based on our results, the reaction was observed to involve either a concerted or sequential reaction mechanism, depending on the configuration of the ionic pair. The rate-limiting step was found to correspond to the nucleophilic attack of the aniline. In addition, the products that were formed depend on which accepting O atom received the proton transferred from the amine N, resulting in two possible reaction mechanisms. The results from the combined ELF/NCI analyses suggest that, for the mechanism where no CO${}_{2}$ was formed (Scheme 1c), the rate-limiting step was suggestive of a late TS. Conversely, for the path where CO${}_{2}$ was formed (Scheme 1d), an early TS structure appeared to be stabilized by the surrounding environment. Additionally, the ELF results suggest that the proton transferred from the aniline in the second stage of the reaction involved a strong H–bond interaction similar to an LBHB for Scheme 1c compared with Scheme 1d, where the proton transfer occurred after the rate-limiting barrier was overcome. The inclusion of explicit polarization of the MM environment was observed to play an important role in the energetics of the MEP and the charge density distribution of the QM subsystem.

## Figures and Tables

**Table 1:** Quantum-mechanics/molecular-mechanics (QM/MM) reaction energies (kcal/mol) corresponding to Scheme 1.

Mechanism	Configuration C1	Configuration C2
a	114.46	27.42
b	174.51	–
c	−27.93	−20.88
d	−21.86	−20.14

**Table 2:** ESP charges for selected atoms for the reaction in DCM(GP) for Scheme 1c.

Atom	Reactant	Transition State	Product
C(1)	0.90(0.89)	0.92(1.02)	0.93(0.93)
O(13)	−0.45(−0.45)	−0.66(−0.66)	−0.60(−0.60)
N(43)	−0.95(−0.94)	−0.76(−0.76)	−0.79(−0.78)
H(44)	0.39(0.39)	0.42(0.43)	0.40(00.39)
H(45)	0.38(0.38)	0.41(0.41)	0.43(00.44)

**Table 3:** ELF population (pop.) analysis for TS structures for the reaction in the GP, DCM, and Configurations C1 and C2 for Scheme 1c.

Basin	GP Pop.	DCM Pop.	C1 TS1 Pop.	C2 TS1 Pop.	C2 TS2 Pop.
V(H44,N43)	2.26	2.24	2.16	2.21	2.17
V(H45,N43)	2.34	2.33	2.48	2.24	–
V(C1,O15)	1.79	1.73	1.69	1.61	1.87
V(C1,O14)	2.50	2.30	2.21	2.00	2.37
V(C1,O13)	–	–	1.23	1.20	–
V(C6,O13)	2.14	2.13	1.89	1.84	2.17
V(C6,O12)	2.68	2.65	2.57	2.64	2.44
V(C1,N43)	2.06	2.07	2.54	2.81	2.06
V(H45)	–	–	–	–	0.51

**Table 4:** ELF populations and first polar moment (μ) analysis for specific basins involved in the reaction in the water/IL mixture corresponding to Configuration C2, Scheme 1d.

Basin	1st TS		MI		2nd TS	
	Pop.	μ (au)	Pop.	μ (au)	Pop.	μ (au)
V(H44,N43)	2.24	1.869	2.18	1.765	2.16	1.64
V(H45,N43)	2.31	1.817	2.18	1.756	–	–
V(H45,O11)	–	–	–	–	1.64	1.35
V(C1,O15)	1.86	0.08	1.93	0.073	1.84	0.041
V(C1,O14)	2.46	0.726	2.4	0.55	2.27	0.237
V(C1,O13)	1.23	0.319	–	–	–	–
V(C1,N43)	–	–	2.00	0.148	2.6	1.085
V(C6,O13)	1.67	0.069	2.09	0.242	2.39	0.577
V(C6,O12)	2.39	0.645	2.13	0.239	2.24	0.393
V(C6,O11)	1.81	0.019	1.58	0.061	1.18	0.294

## Supplementary Materials

The following are available online at http://www.mdpi.com/1420-3049/23/11/2830/s1: Energy interaction analysis for parametrized fragments, additional MEP, ESP, ELF/NCI, ELF populations and reaction path animations.

## Abbreviations

The following abbreviations are used in this manuscript:

IL	Ionic liquids
RTIL	Room temperature ionic liquid
EMIm	1–Ethyl–3–methylimidazolium
BF${}_{4}$	Tetrafluoroborate
QM	Quantum Mechanical
MM	Molecular Mechanical
MD	Molecular Dynamics
NPFF	Non–polarizable force fields
PFF	Polarizable force fields
GEM	Gaussian Electrostatic Model
TS	Transition State
RC	Reaction Coordinate
GP	Gas–phase
DCM	Dichloromethane
ELF	Electron Localization Function
NCI	Noncovalent Interaction
BFGS	Broyden–Fletcher–Goldfarb–Shanno
