# Supplementary figures and images for: Polarizable ab initio QM/MM Study of the Reaction Mechanism of N-tert-Butyloxycarbonylation of Aniline in [EMIm][BF4]
Source: Molecules. 2018 Oct 31;23(11):2830. doi: 10.3390/molecules23112830 (PMC6278528; doi:10.3390/molecules23112830)

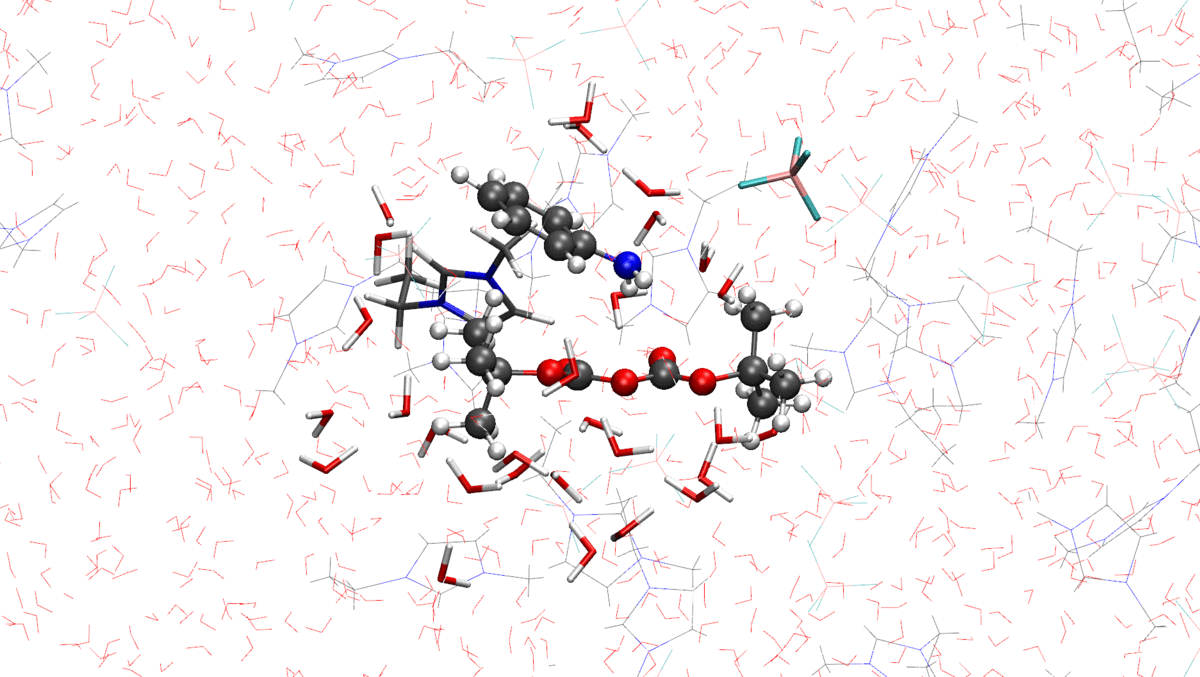

Supplement: Supplementary file 1 [file molecules-23-02830-s001.zip › Si-polarizable-ab-initio/VideoS5.gif]

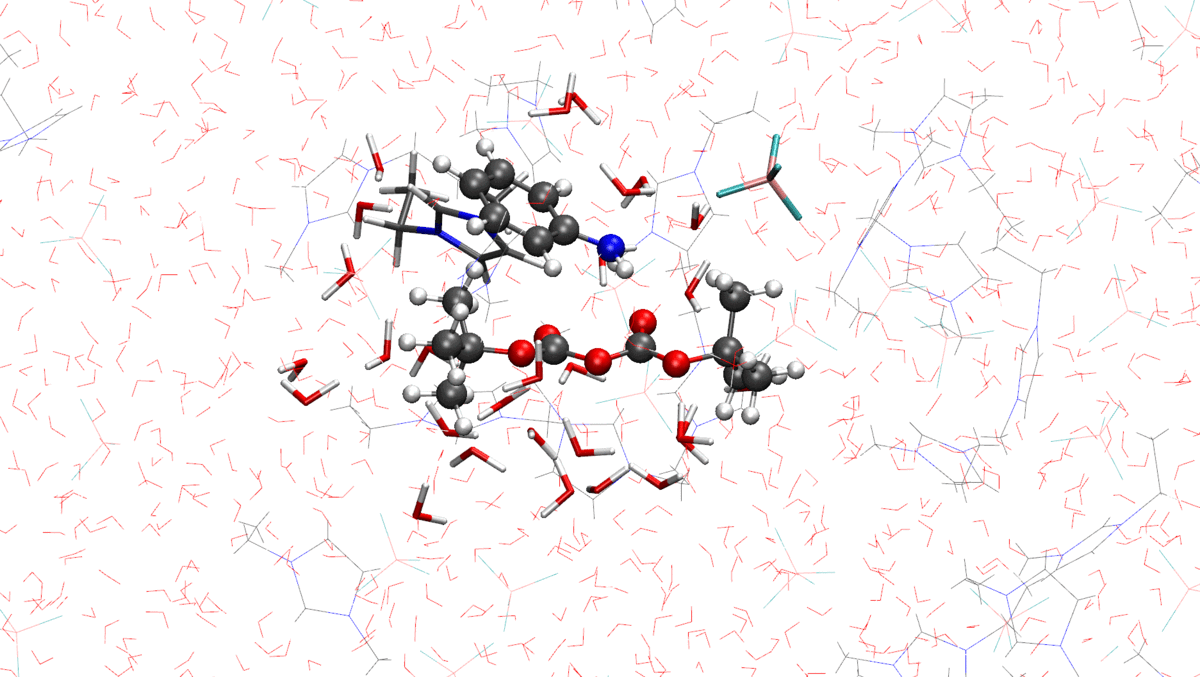

Supplement: Supplementary file 1 [file molecules-23-02830-s001.zip › Si-polarizable-ab-initio/VideoS4.gif]

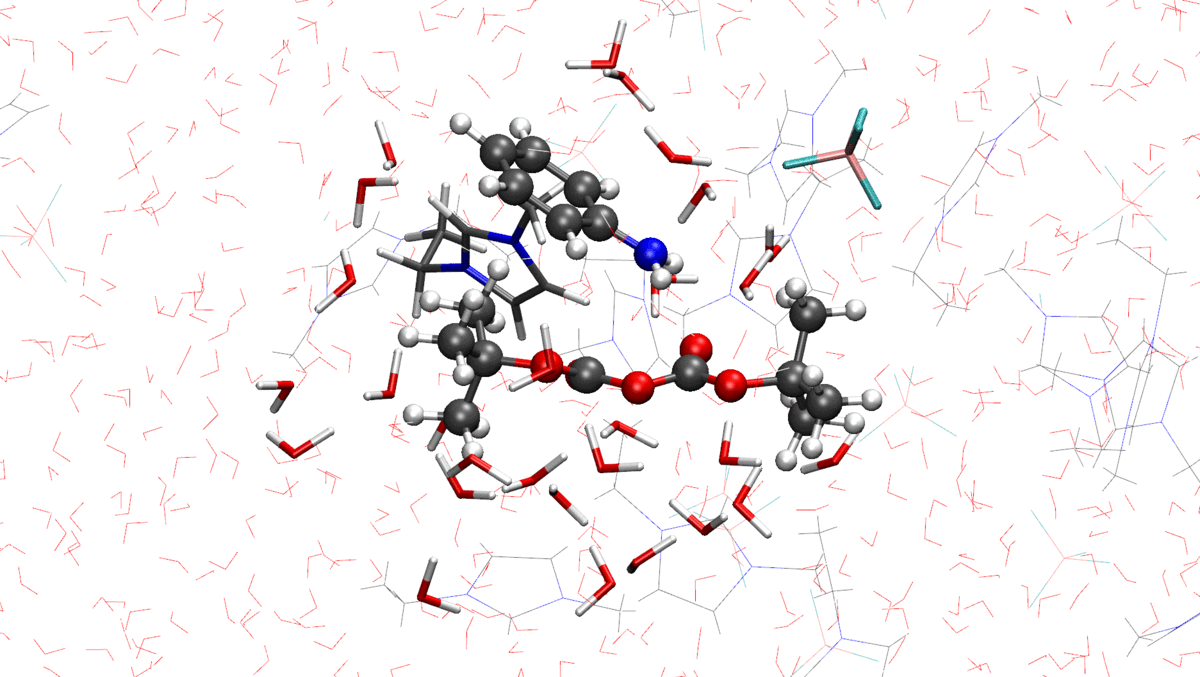

Supplement: Supplementary file 1 [file molecules-23-02830-s001.zip › Si-polarizable-ab-initio/VideoS3.gif]

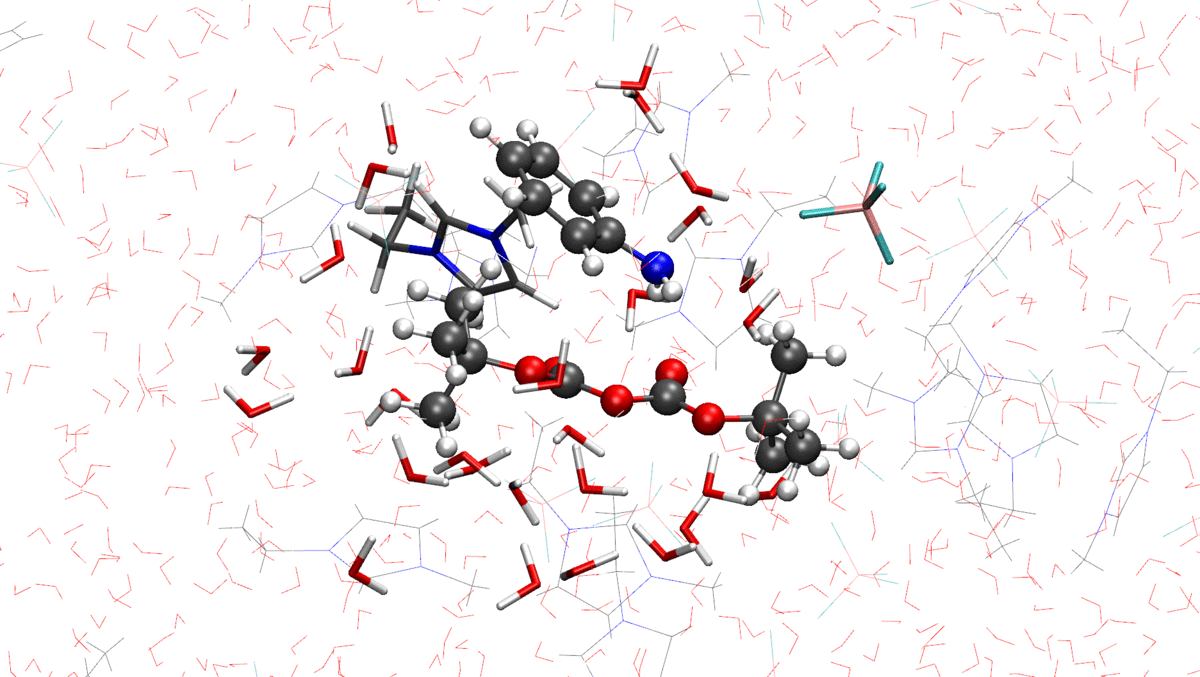

Supplement: Supplementary file 1 [file molecules-23-02830-s001.zip › Si-polarizable-ab-initio/VideoS2.gif]

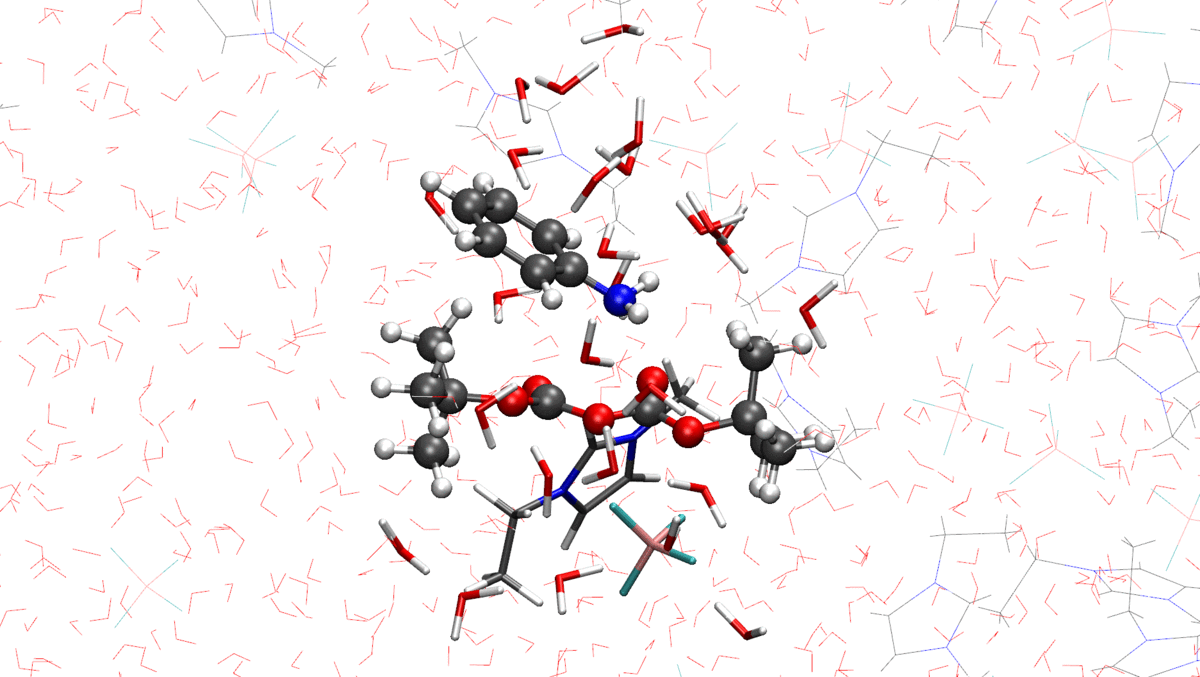

Supplement: Supplementary file 1 [file molecules-23-02830-s001.zip › Si-polarizable-ab-initio/VideoS1.gif]

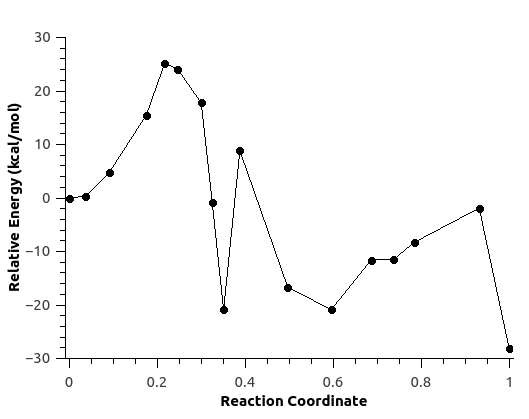

Supplement: Supplementary file 1 [file molecules-23-02830-s001.zip › Si-polarizable-ab-initio/images/MEP-C1-2C.png]

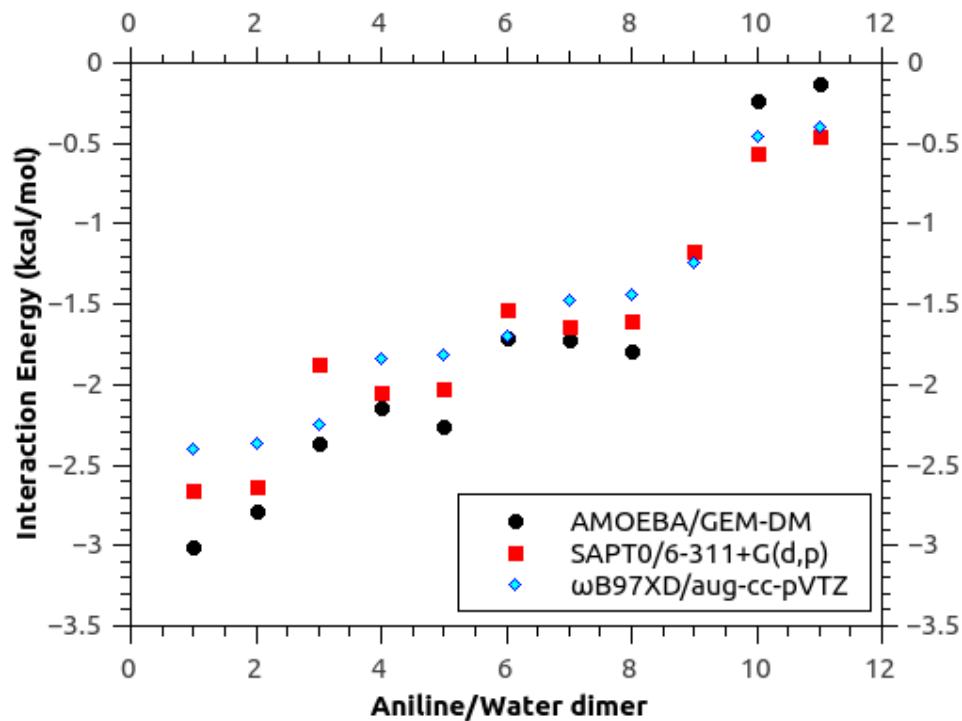

Supplement: Supplementary file 1 [file molecules-23-02830-s001.zip › Si-polarizable-ab-initio/images/aniline.pdf]

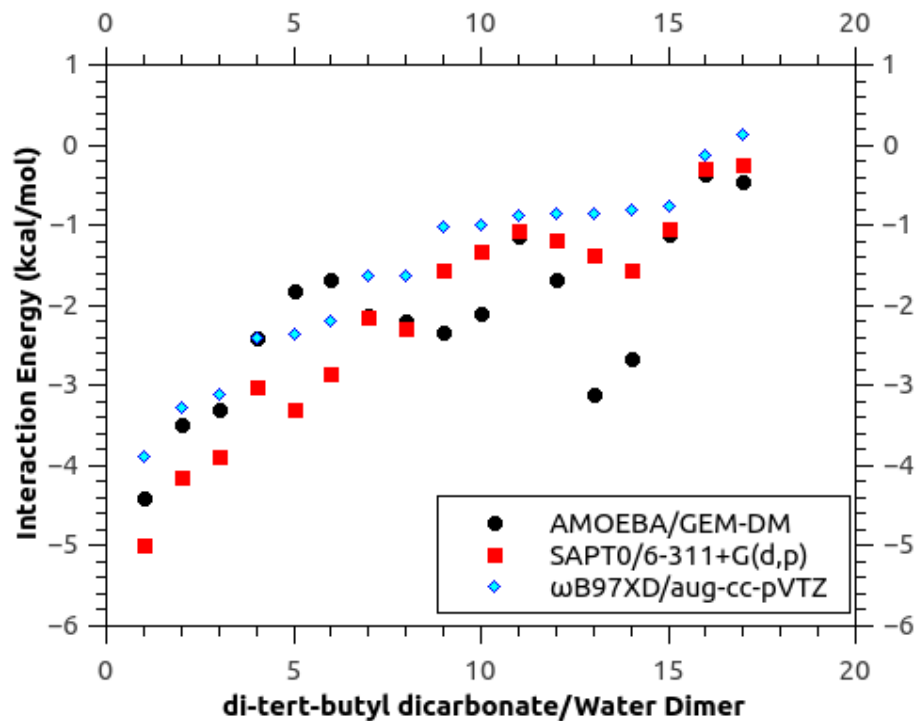

Supplement: Supplementary file 1 [file molecules-23-02830-s001.zip › Si-polarizable-ab-initio/images/db.pdf]

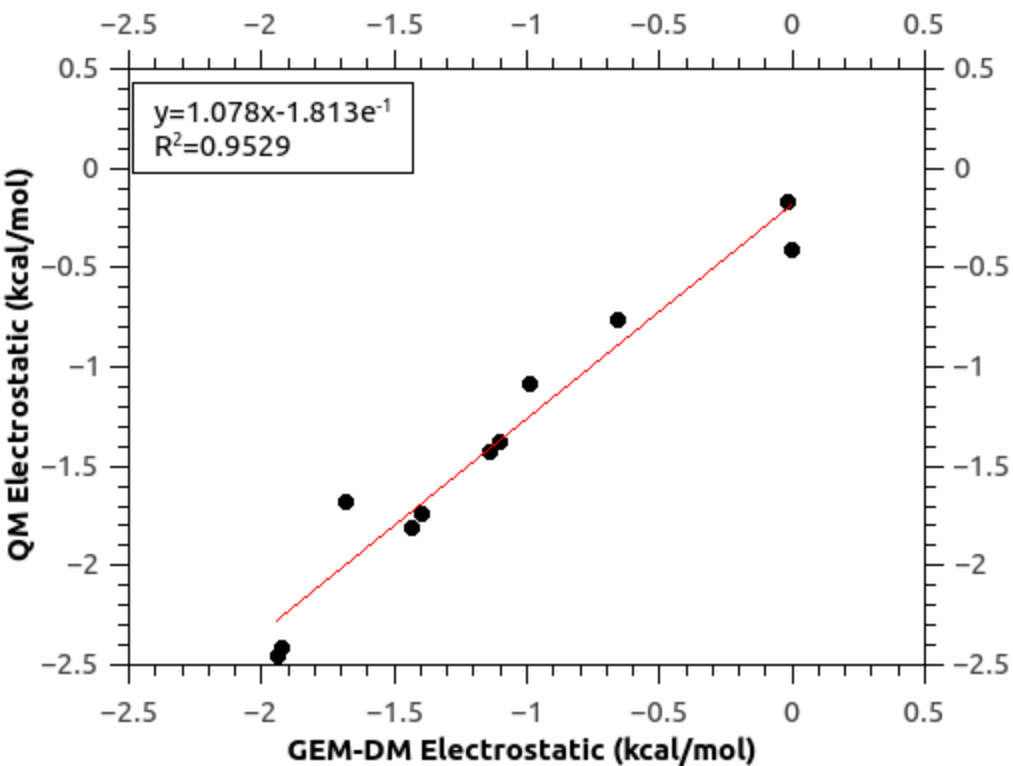

Supplement: Supplementary file 1 [file molecules-23-02830-s001.zip › Si-polarizable-ab-initio/images/aniline2.pdf]

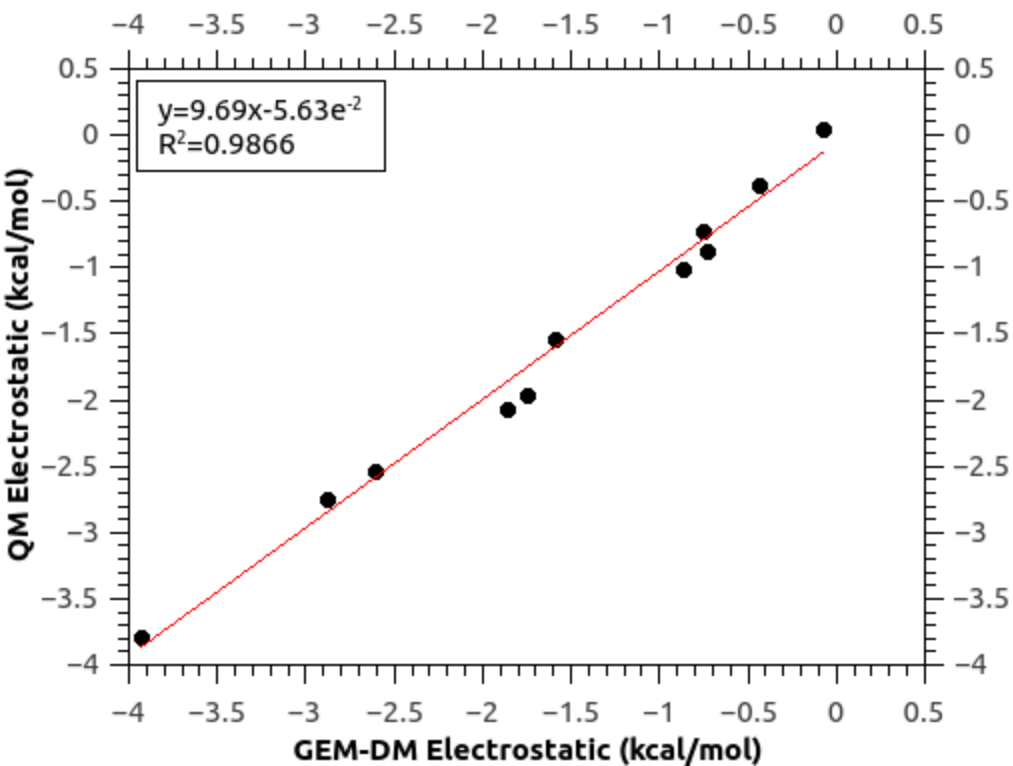

Supplement: Supplementary file 1 [file molecules-23-02830-s001.zip › Si-polarizable-ab-initio/images/db2.pdf]

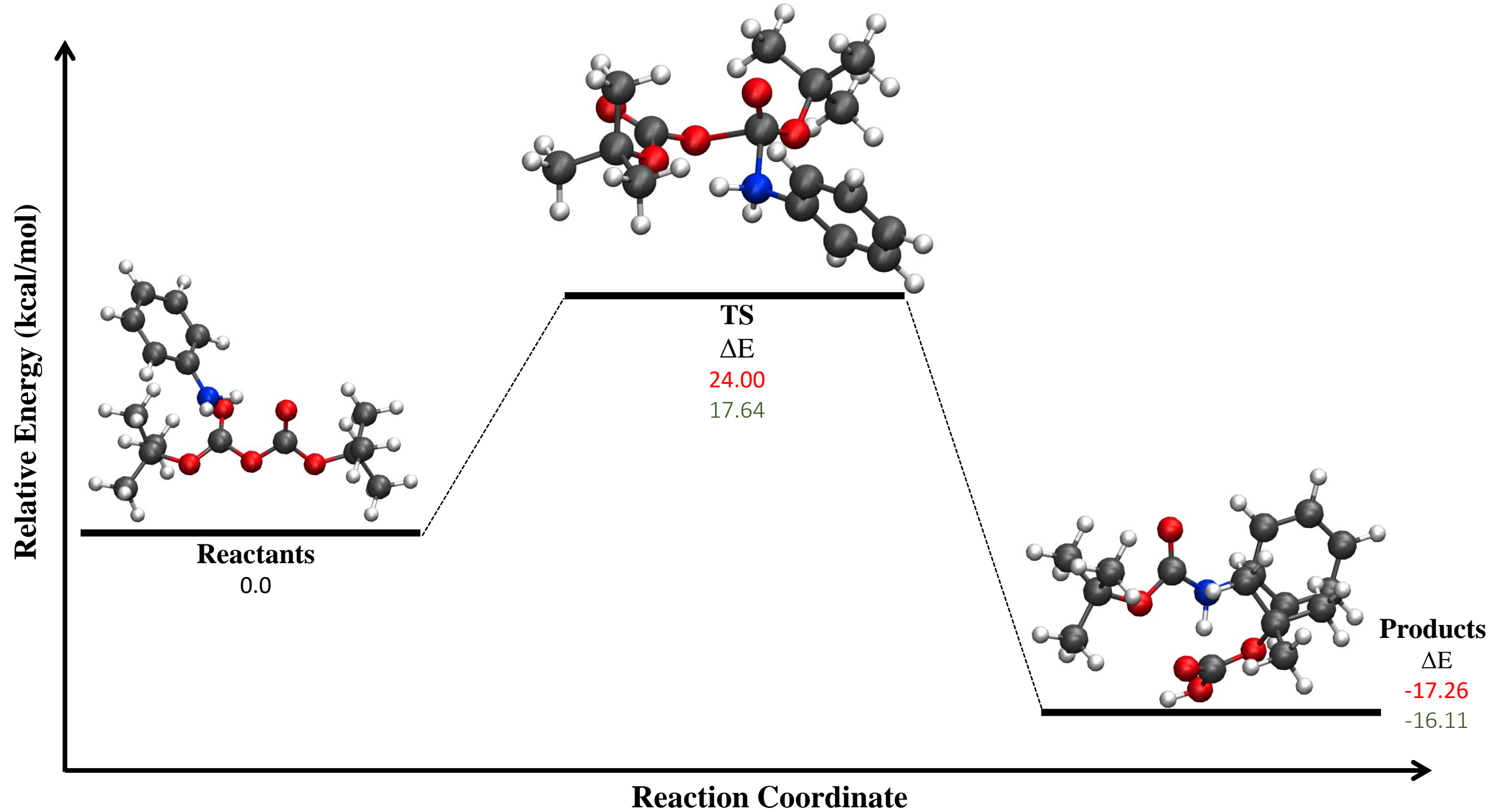

Supplement: Supplementary file 1 [file molecules-23-02830-s001.zip › Si-polarizable-ab-initio/images/rxn-path.pdf]

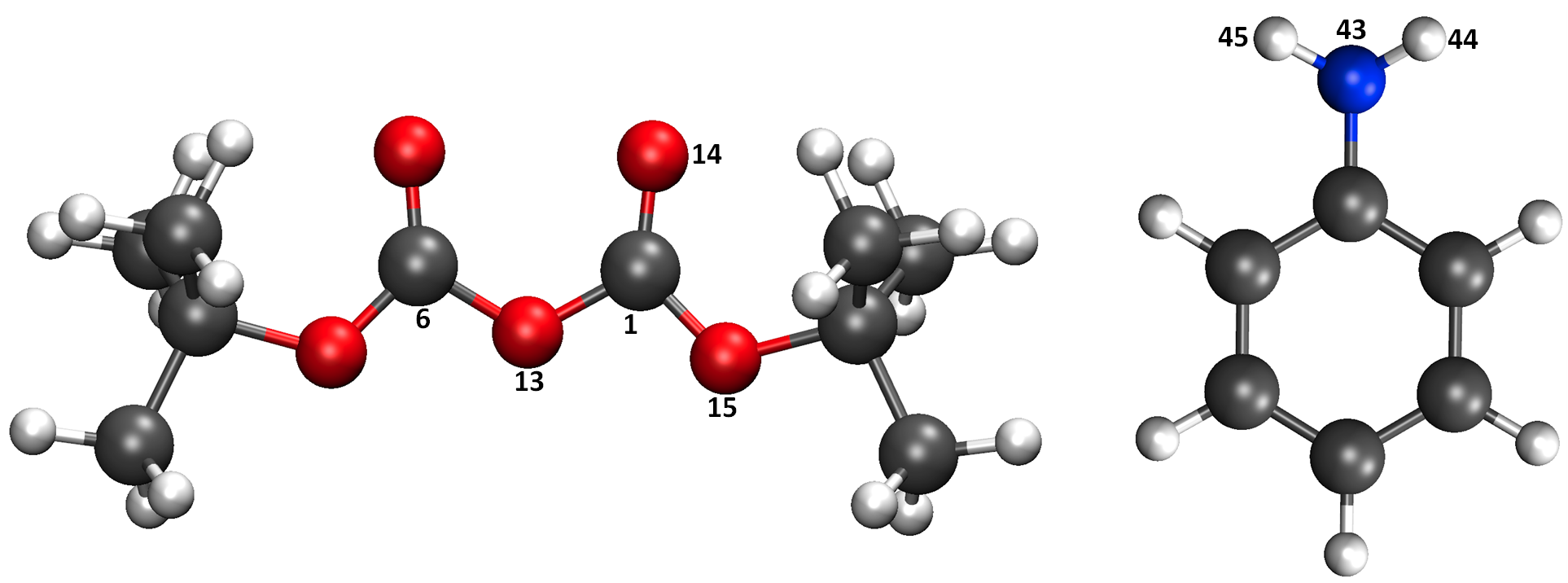

Supplement: Supplementary file 1 [file molecules-23-02830-s001.zip › Si-polarizable-ab-initio/images/reactants.png]

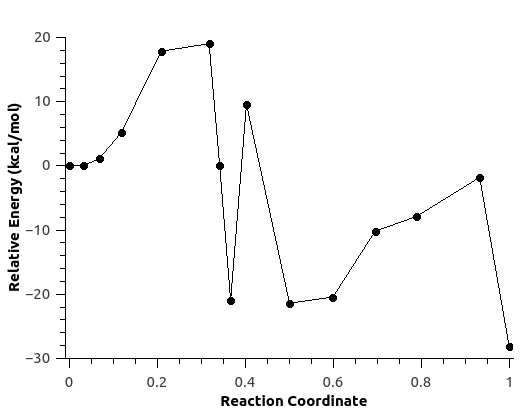

Supplement: Supplementary file 1 [file molecules-23-02830-s001.zip › Si-polarizable-ab-initio/images/MEP-C1.png]

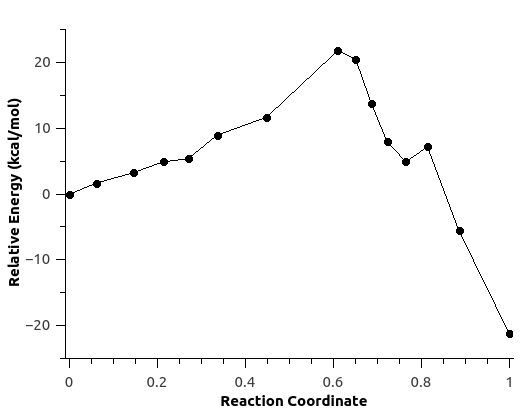

Supplement: Supplementary file 1 [file molecules-23-02830-s001.zip › Si-polarizable-ab-initio/images/MEP-C2.png]

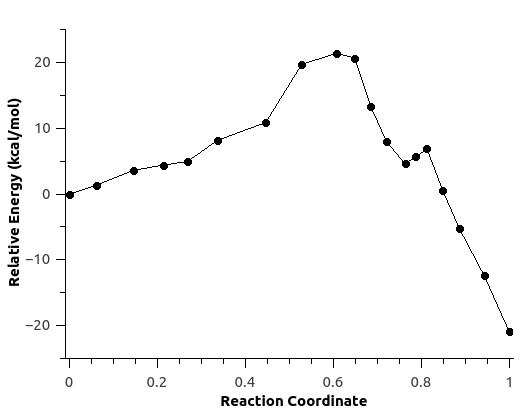

Supplement: Supplementary file 1 [file molecules-23-02830-s001.zip › Si-polarizable-ab-initio/images/MEP-C2-2.png]

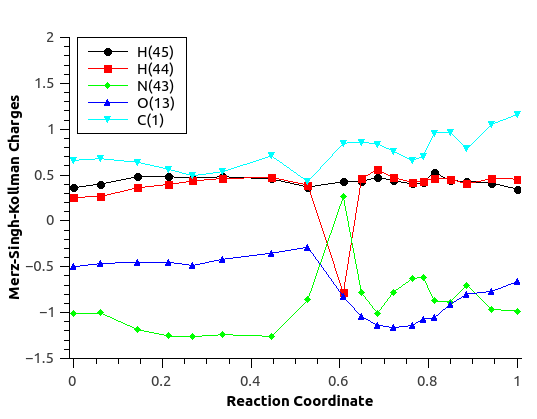

Supplement: Supplementary file 1 [file molecules-23-02830-s001.zip › Si-polarizable-ab-initio/images/MK-charges-C2-2.png]

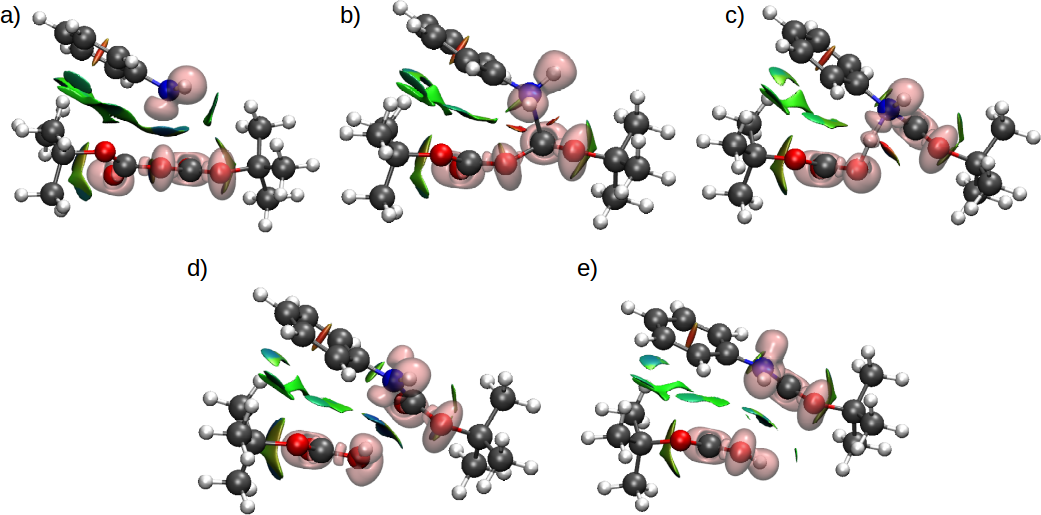

Supplement: Supplementary file 1 [file molecules-23-02830-s001.zip › Si-polarizable-ab-initio/images/IL.png]

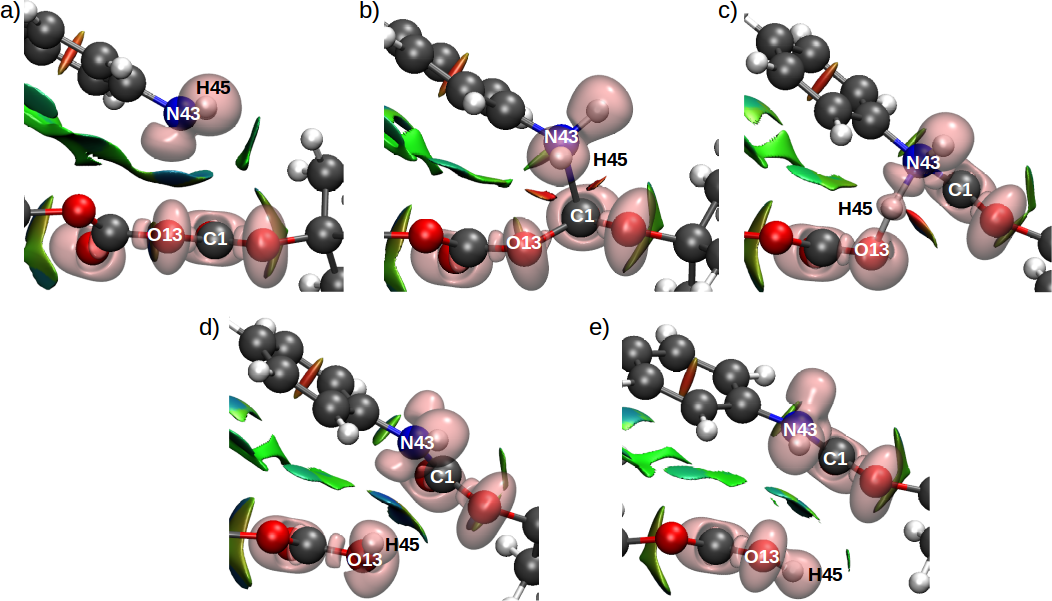

Supplement: Supplementary file 1 [file molecules-23-02830-s001.zip › Si-polarizable-ab-initio/images/ILzoom.png]

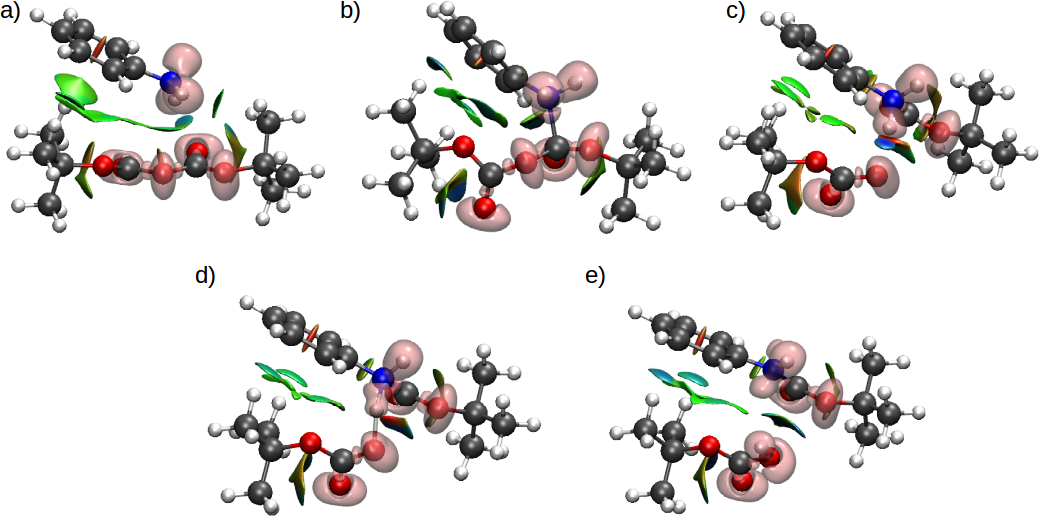

Supplement: Supplementary file 1 [file molecules-23-02830-s001.zip › Si-polarizable-ab-initio/images/ILnew.png]

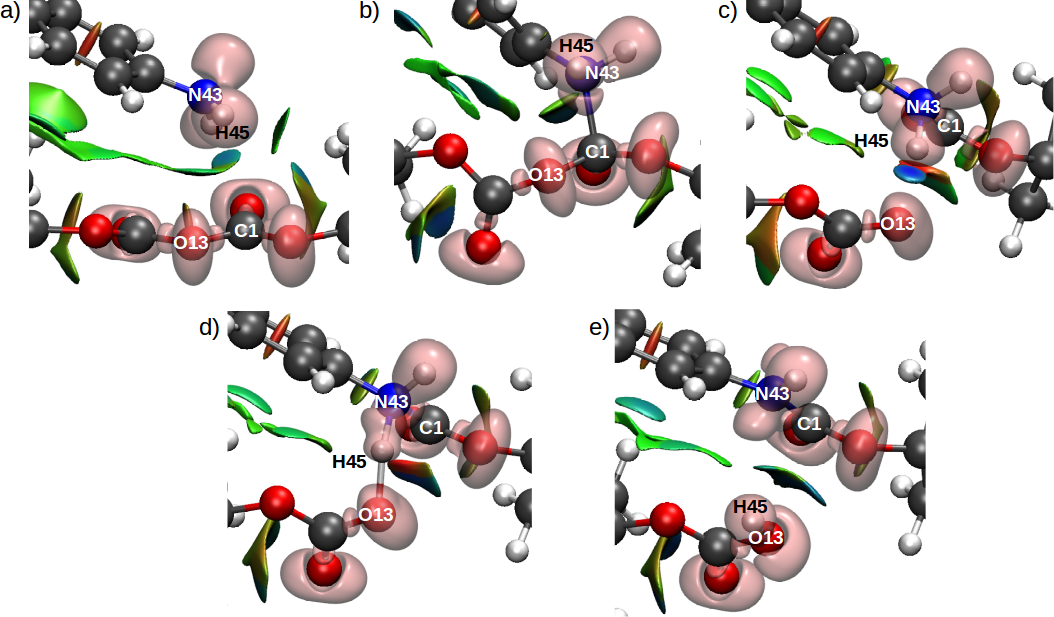

Supplement: Supplementary file 1 [file molecules-23-02830-s001.zip › Si-polarizable-ab-initio/images/ILnewzoom.png]

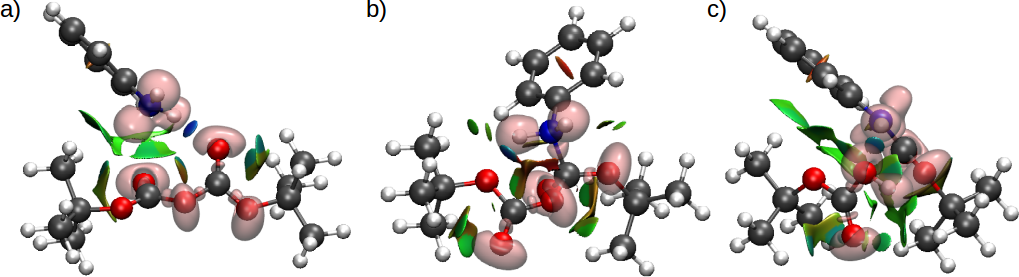

Supplement: Supplementary file 1 [file molecules-23-02830-s001.zip › Si-polarizable-ab-initio/images/dcmnew.png]

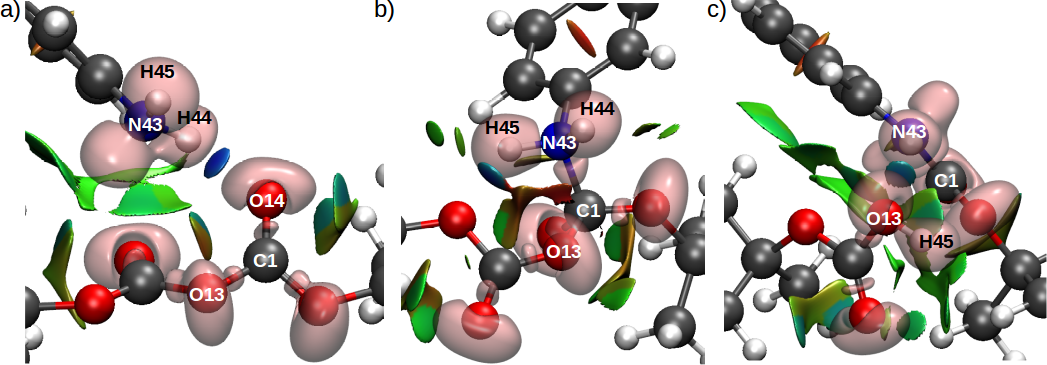

Supplement: Supplementary file 1 [file molecules-23-02830-s001.zip › Si-polarizable-ab-initio/images/dcmnewzoom.png]

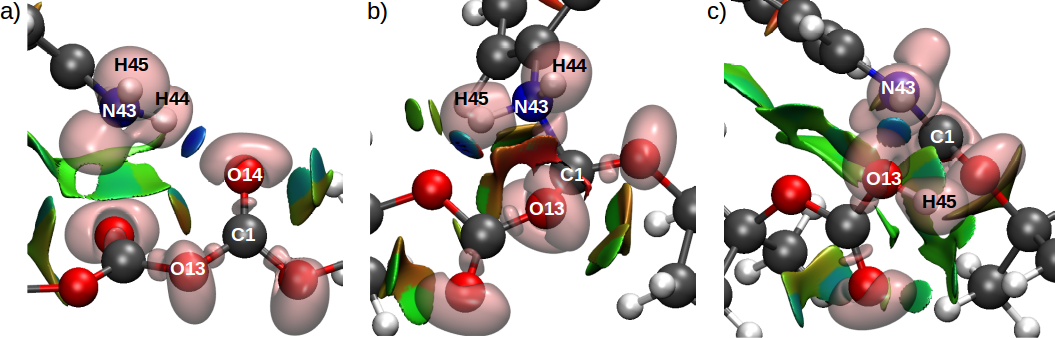

Supplement: Supplementary file 1 [file molecules-23-02830-s001.zip › Si-polarizable-ab-initio/images/gpnewzoom.png]

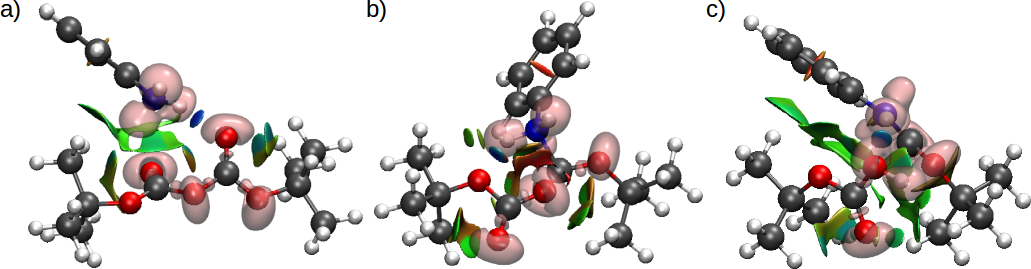

Supplement: Supplementary file 1 [file molecules-23-02830-s001.zip › Si-polarizable-ab-initio/images/gpnew.png]

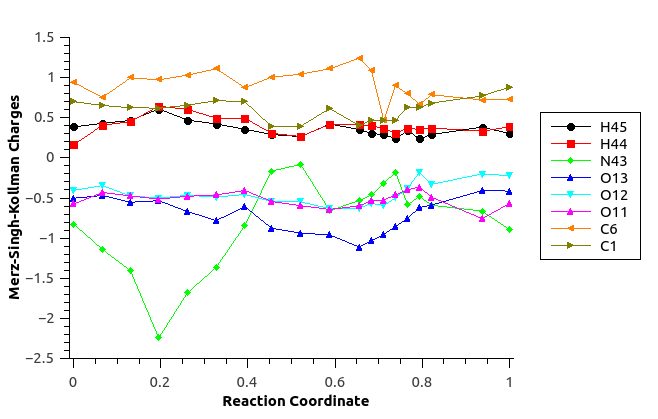

Supplement: Supplementary file 1 [file molecules-23-02830-s001.zip › Si-polarizable-ab-initio/images/MK-charges-C2-CO2.png]

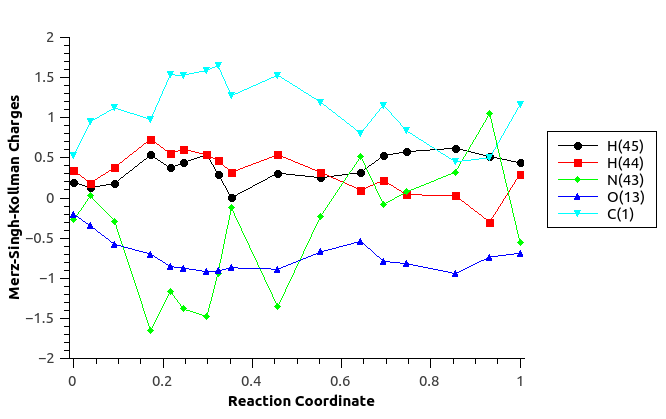

Supplement: Supplementary file 1 [file molecules-23-02830-s001.zip › Si-polarizable-ab-initio/images/MK-charges-C1-2.png]

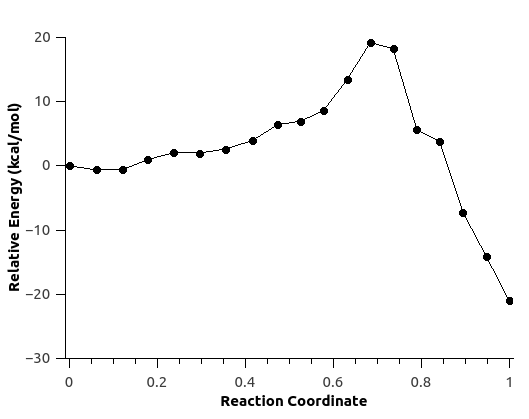

Supplement: Supplementary file 1 [file molecules-23-02830-s001.zip › Si-polarizable-ab-initio/images/MEP-C2-QSM.png]

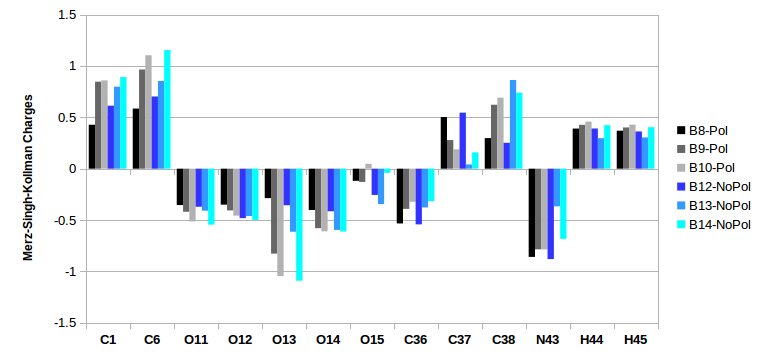

Supplement: Supplementary file 1 [file molecules-23-02830-s001.zip › Si-polarizable-ab-initio/images/MK-comparison-C.png]

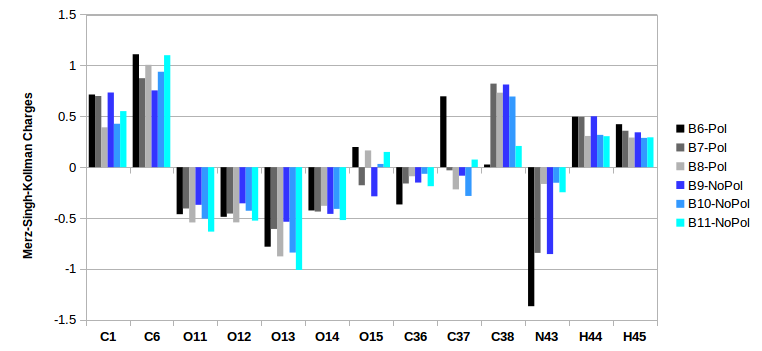

Supplement: Supplementary file 1 [file molecules-23-02830-s001.zip › Si-polarizable-ab-initio/images/MK-comparison-D.png]
